# Epidemiology of combined clavicle and rib fractures: a systematic review

**DOI:** 10.1007/s00068-021-01701-4

**Published:** 2021-06-01

**Authors:** Arthur A. R. Sweet, Reinier B. Beks, Frank F. A. IJpma, Mirjam B. de Jong, Frank J. P. Beeres, Luke P. H. Leenen, Roderick M. Houwert, Mark C. P. M. van Baal

**Affiliations:** 1 Department of Surgery, University Medical Center Utrecht, 85500, 3508 GA Utrecht, The Netherlands; 2 Department of Surgery, Noordwest Ziekenhuisgroep, Alkmaar, The Netherlands; 3 Department of Surgery, University Medical Center Groningen, Groningen, The Netherlands; 4 Department of Orthopedics and Trauma Surgery, Luzerner Kantonsspital, Luzern, Switzerland

**Keywords:** Clavicle fractures, Rib fractures, Epidemiology, Treatment

## Abstract

**Purpose:**

The aim of this systematic review was to provide an overview of the incidence of combined clavicle and rib fractures and the association between these two injuries.

**Methods:**

A systematic literature search was performed in the MEDLINE, EMBASE, and CENTRAL databases on the 14^th^ of August 2020. Outcome measures were incidence, hospital length of stay (HLOS), intensive care unit admission and length of stay (ILOS), duration of mechanical ventilation (DMV), mortality, chest tube duration, Constant–Murley score, union and complications.

**Results:**

Seven studies with a total of 71,572 patients were included, comprising five studies on epidemiology and two studies on treatment. Among blunt chest trauma patients, 18.6% had concomitant clavicle and rib fractures. The incidence of rib fractures in polytrauma patients with clavicle fractures was 56–60.6% versus 29% in patients without clavicle fractures. Vice versa, 14–18.8% of patients with multiple rib fractures had concomitant clavicle fractures compared to 7.1% in patients without multiple rib fractures. One study reported no complications after fixation of both injuries. Another study on treatment, reported shorter ILOS and less complications among operatively versus conservatively treated patients (5.4 ± 1.5 versus 21 ± 13.6 days).

**Conclusion:**

Clavicle fractures and rib fractures are closely related in polytrauma patients and almost a fifth of all blunt chest trauma patients sustain both injuries. Definitive conclusions could not be drawn on treatment of the combined injury. Future research should further investigate indications and benefits of operative treatment of this injury.

**Supplementary Information:**

The online version contains supplementary material available at 10.1007/s00068-021-01701-4.

## Introduction

Thoracic injuries are one of the main causes of death, both in isolated chest trauma patients as well as in polytrauma patients [1, 2]. Blunt thoracic trauma contributes to complications and mortality as it may directly injure vital thoracic and abdominal structures secured by the chest wall, but also secondarily by impairing the chest wall integrity [3–6]. Both clavicle fractures and rib fractures have been shown to act as a marker of severity of the chest injury and have both independently been shown to increase the risk of mortality [7–11]. A combination of clavicle and rib fractures may further worsen the outcome. Literature underlines the impact of combined clavicle fractures and multiple upper rib fractures, as it may lead to severe thoracic deformities and loss of function of the shoulder [12].  Furthermore, ipsilateral chest wall injuries have been shown to contribute to secondary displacement of the clavicle fracture, especially in patients with upper rib fractures [13, 14].

In polytrauma patients who suffered a blunt chest trauma, rib fractures are the most prevalent chest injuries, followed by intra-thoracic injuries and clavicle fractures [15]. Rib fractures are mostly treated conservatively with pain control, mobilization and pulmonary care. However, several recent studies have shown benefits of operative treatment of multiple displaced rib fractures and flail chest injuries, compared to conservative treatment [16–19]. More than 10% of polytrauma patients suffer from a clavicle fracture, with 77% of those also sustaining other thoracic injuries [9]. Treatment of isolated clavicle fractures primarily depends on the location, displacement, and degree of comminution of the fracture [20, 21].

Treatment of both injuries has been well described in recent literature as separate entities. Yet, it remains unclear how these two injuries are associated with each other and whether these injuries should be managed differently if they occur at the same time. Therefore, this study primarily aims to provide an overview of all literature that is available on the incidence of combined clavicle and rib fractures and on the association between these two injuries. Secondarily, all studies on treatment and outcomes of patients with this combined injury will be assessed.

## Methods

In this systematic review, the Preferred Reporting Items for Systematic Reviews and Meta-Analyses (PRISMA) guideline was followed [22]. A protocol of this systematic review has not been published.

Eligibility criteria were all studies that reported on patients with combined injuries of clavicle and rib fractures. Exclusion criteria were studies on patients under the age of 16 years, languages other than English, German or Dutch and case reports. There were no restrictions on publication dates. A broad literature search was performed for studies reporting on patients with both clavicle and rib fractures in the MEDLINE, EMBASE, and CENTRAL (Cochrane Central Register of Controlled Trials) databases on the 14^th^ of August 2020. The inclusion of studies was discussed between two reviewers (AS and RB). The complete search terms syntax is written in Appendix 1. References and citations of all included studies were screened for other eligible studies.

Data were extracted using a data extraction file, including study design, study population, number of patients, age, sex, Injury Severity Score (ISS), mechanism of trauma (blunt or penetrating), number of patients with clavicle fractures and number of patients with rib fractures. Outcome measures were hospital length of stay (HLOS), intensive care unit admission, intensive care unit length of stay (ILOS), number of patients who needed mechanical ventilation, duration of mechanical ventilation (DMV), number of patients treated with a chest tube, mortality, whether the patient had surgery of the clavicle and ribs, duration until surgery, chest tube duration, Constant–Murley score [23], complete union of the fixated fractures and complications.

The methodological index for non-randomized studies (MINORS), a validated instrument to assess the methodological quality of non-randomized surgical studies, was used to assess the methodological quality of the included studies [24]. The MINORS score ranges from 0 to 24, with higher scores representing better methodological quality. The complete MINORS scores of all included studies are noted in Online Appendix 2.

Studies were described separately for two different subjects using descriptive statistics. Dichotomous variables were presented as numbers with proportions. Continuous variables were given as mean ± standard deviation (SD) in case of a normal distribution and as median and interquartile range (IQR) in case of a non-normal distribution. First, all studies on the epidemiology of combined clavicle and rib fractures were reported. Second, the studies that reported on operative treatment of patients who sustained both clavicle fractures and rib fractures were presented.

## Results

**Fig. 1:**
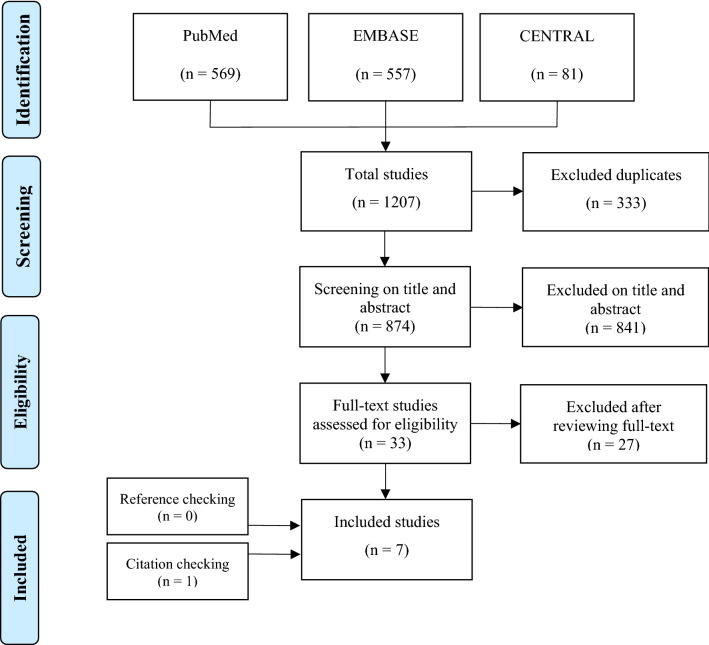
PRISMA flow diagram representing the search and screen process of articles describing patients with combined clavicle and rib fractures

A total of seven studies were included in this review (Fig. 1). Five studies on a total of 71,572 patients reported on epidemiological data of clavicle fractures and rib fractures and two studies on 27 patients reported on operative treatment and outcomes of these combined injuries [25–31].

### The epidemiology of combined clavicle and rib fractures

One retrospective cohort study reported on 1621 consecutive blunt thoracic trauma patients with a mean age of 51.2 ± 17.1 years and 6.9% with an ISS ≥ 16 (Table 1) [25]. In total, 21.5% had a clavicle fracture, 78.5% had rib fractures, and 18.6% sustained both injuries. Multivariate logistic regression analysis showed that clavicle fractures were significantly associated with the presence of rib fractures with an odds ratio of 1.68 (CI 1.19–2.37).

**Table 1:** Characteristics and outcomes of included studies on the association and incidence of patients with clavicle fractures and rib fractures

Study	Study design	Country	Study population	Patients *n*	Age in years mean ± SD	Male *n* (%)	ISS mean ± SD	ISS > 16 *n* (%)	Blunt mechanism of trauma *n* (%)	Rib fractures *n* (%)	Clavicle fractures *n* (%)	HLOS days (IQR)	ICU admission *n* (%)	ILOS days ± SD	Mechanical ventilation *n* (%)	DMV days ± SD	Chest tube *n* (%)	Mortality *n* (%)
Lin et al. 2016	Retrospective cohort	Taiwan	Blunt thoracic trauma	1621	51.2 ± 17.1	1176 (72.5)	NR	190 (6.9)	1621 (100)	1272 (78.5)	349 (21.5)	NR	NR	NR	NR	NR	420 (25.9)	112 (6.9)
Horst et al. 2015	Retrospective cohort	Germany	Clavicle fracture	4790	47 ± 19	3501 (73.1)	30 ± 11	4790 (100)	4713 (98.4)	2682 (56)	4790 (100)	25 ± 25	NR	12 ± 14	NR	7.4 ± 11.2	NR	599 (12.5)
			No clavicle fracture	41,775	48 ± 21	30496 (73.0)	28 ± 12	41,775 (100)	40,020 (95.8)	12115 (29)	0 (0)	24 ± 26	NR	10 ± 13	NR	6.3 ± 11.0	NR	7895 (18.9)
Van Laarhoven et al. 2016	Retrospective cohort	Netherlands	Clavicle fracture	160	47.5 ± 20.9	107 (66.9)	29.2 ± 10.1	160 (100)	160 (100)	97 (60.6)	160 (100)	21.5 (5-30)	NR	NR	72 (45)	NR	46 (29)	35 (21.9)
			No clavicle fracture	1301	49.2 ± 21.6	915 (70.3)	24.9 ± 9.1	1301 (100)	1301 (100)	378 (29.1)	0 (0)	16.5 (3 - 21)	NR	NR	NR	NR	NR	231 (17.8)
Hashemzadeh et al. 2018	Cross-sectional study	Iran	Upper rib fractures	8	51.5 ± 12.47	8 (100)	25.70 ± 8.82	NR	8 (100)	8 (100)	4 (50)	32.75 ± 25.57	4 (50)	11.77 ± 1.79	4 (50)	NR	8 (100)	NR
			Middle rib fractures	170	46.63 ± 15.46	138 (81.2)	16.98 ± 10.24	NR	170 (100)	170 (100)	22 (12.9)	9.19 ± 6.69	38 (22.4)	11.77 ± 1.79	18 (10.6)	NR	118 (69.4)	NR
			Lower rib fractures	6	37.00 ± 0.89	6 (100)	17.66 ± 7.22	NR	6 (100)	6 (100)	0 (0)	10.33 ± 6.94	2 (33.3)	11.77 ± 1.79	0 (0)	NR	4 (66.7)	NR
Schulz-Drost et al. 2016	Retrospective cohort	Germany	No rib fractures	11,267	42.8 ± 21.8	8101 (71.9)	27.6 ± 11.6	11,267 (100)	10,546 (93.6)	0 (0)	800 (7.1)	21.5 ± 21.1	10,174 (90.3)	9.0 ± 12.3	NR	4.9 ± 9.0	1161 (10.3)	1949 (17.3)
			1 Rib fracture	919	NR	NR	NR	919 (100)	NR	919 (100)	130 (14.1)	NR	NR	NR	NR	NR	NR	NR
			2 Rib fractures	1038	NR	NR	NR	1038 (100)	NR	1038 (100)	169 (16.3)	NR	NR	NR	NR	NR	NR	NR
			≥3 Rib fractures	5025	51.7 ± 19.4	3668 (73.0)	28.3 ± 11.3	5025 (100)	4970 (98.9)	5025 (100)	958 (19.1)	22.6 ± 20.4	4638 (92.3)	10.6 ± 13.4	NR	5.9 ± 10.1	1447 (28.8)	734 (14.6)
			Flail chest	3492	54.1 ± 18.2	2619 (75.0)	35.1 ± 14.2	3492 (100)	3436 (98.4)	3492 (100)	714 (20.4)	23.7 ± 22.7	3087 (88.4)	12.3 ± 15.1	NR	7.3 ± 11.9	1585 (45.4)	803 (23.0)

*SD* standard deviation, *ISS* injury severity score, *HLOS* hospital length of stay, *ICU* intensive care unit, *ILOS* intensive care unit length of stay, *DMV* duration of mechanical ventilation, *NR* not reported

Two retrospective cohort studies on polytrauma patients compared the incidence of rib fractures in patients with or without clavicle fractures [26, 27]. One study on data of 46,565 patients from the Trauma Register DGU (Deutsche Gesellschaft für Unfallchirurgie) from 2002 until 2011 included all patients with rib fractures who were above 16 years of age with an ISS ≥ 16 (Table 1) [26]. There were 4790 patients with clavicle fractures with a mean age of 47 ± 19 years and 41,775 patients without clavicle fractures with a mean age of 48 ± 21 years. Concomitant rib fractures were significantly more prevalent among patients with clavicle fractures compared to patients without clavicle fractures (56 versus 29%, *P* < 0.001). The second study conducted on data of the Dutch Trauma Registry from 2007 until 2011, included all patients (*n* = 1461) above 18 years of age with an ISS ≥ 16 (Table 1) [27]. This study reported on 160 patients with clavicle fractures with a mean age of 47.5 ± 20.9 and 1301 patients without clavicle fractures with a mean age of 49.2 ± 21.6. There were significantly more patients with rib fractures among patients with clavicle fractures compared to patients without clavicle fractures (60.6 versus 29.1%, *P* < 0.001).

Two studies reported on the incidence of clavicle fractures in patients with rib fractures following blunt chest trauma [28, 29]. One study investigated 184 patients with rib fractures with a mean age of 46.5 ± 15.2 years and 45.7% had an ISS ≥ 16 (Table 1) [28]. A total of 14% of these patients also suffered from a concomitant clavicle fracture. Subgroups of patients with upper (1–2), middle (3–8), and lower (9–12) rib fractures were compared and the number of clavicle fractures was found to be significantly higher in patients with upper rib fractures as compared to middle and lower rib fractures (50 versus 12.9% versus 0%, *P* < 0.001). A retrospective cohort study on data of 21,741 polytrauma patients with an ISS ≥ 16 from the Trauma Registry DGU from 2009 until 2013 compared patients with multiple (≥ 3) rib fractures (*n* = 5025) with patients with flail chests (*n* = 3492) and a control group of patients without rib fractures (*n* = 11,267) (Table 1) [29]. Concomitant clavicle fractures were seen in 18.8% of the patients with rib fractures or flail chests compared to 7.1% of the patients without rib fractures. The percentages of clavicle fractures were 14.1%, 16.3%, 19.1%, and 20.4% in patients with one, two, three rib fractures, and a flail chest, respectively.

The average MINORS score of the comparative studies was 17.5 ± 1 (17–19) and the non-comparative study had a MINORS score of 11 (Online Appendix 2).

### Operative treatment and outcomes of patients with combined clavicle and rib fractures

One prospective case series study investigated 11 blunt chest trauma patients with a mean age of 58.5 ± 9.2 years who sustained flail chests and clavicle fractures [30]. All patients had operative treatment of both injuries during the same session using a clavipectoral approach and the mean HLOS was 18.8 ± 8.1 days (Table 2). After 12 weeks, two patients still reported painful restriction in movement of the shoulder. After a follow-up of 12 months, all patients had radiological union of the fixated clavicle fractures and rib fractures and no complications were reported. The MINORS score of this non-comparative study was 13 (Online Appendix 2).

**Table 2:** Characteristics and outcomes of included studies on treatment of patients with clavicle fractures and flail chests

Study	Study design	Country	Study population	Patients *n*	Age in years mean ± SD	Male *n* (%)	ISS mean ± SD	Blunt mechanism of trauma *n* (%)	Surgery of ribs *n* (%)	Surgery of clavicle *n* (%)	Follow-up months mean ± SD	Duration until surgery days mean ± SD	HLOS days mean ± SD	ILOS days mean ± SD	DMV days mean ± SD	Chest tube duration days mean ± SD	Constant–Murley score mean ± SD	Union *n* (%)	Complications *n* (%)
Langenbach et al. 2017	Prospective case series	Germany	Operative treatment	11	58.5 ± 9.2	8 (72.7)	NR	11 (100)	11(100)	11 (100)	12	5.8 ± 2.8	18.8 ± 8.1	NR	NR	NR	NR	11 (100)	0 (0)
Solberg et al. 2009	Retrospective case series	USA	Operative treatment	9	38.8 ± 16.7	6 (66.6)	24.9 ± 6.5	9 (100)	9 (100)	7 (77.8)	16.1 ± 6.7	18 (6–42) hours	NR	5.4 ± 1.5	1.9 ± 1.1	5.6 ± 1.2	87.6 ± 5.4	7 (77.8)	2 (22.2)
			Conservative treatment	7	41.1 ± 13.0	5 (71.4)	24.8 ± 6.2	7 (100)	0 (0)	0 (0)	12.0 ± 2.3	n/a	NR	21 ± 13.6	13.3 ± 5.3	16.8 ± 9.75	74.6 ± 9.75	7 (100)	5 (71.4)

*SD* standard deviation, *ISS* injury severity score, *HLOS* hospital length of stay, *ILOS* intensive care unit length of stay, *DMV* duration of mechanical ventilation

A retrospective case series study on 16 blunt chest trauma patients with chest wall injuries caused by side impact mechanisms, leading to clavicle fractures and multiple posterolateral segmental rib fractures, compared outcomes of operative (*n* = 9) versus conservative (*n* = 7) treatment of the rib fractures (Table 2) [31]. Seven out of nine patients in the operative group also had operative treatment of the clavicle fracture. Comparing the operative with the conservative group, the ILOS (5.4 ± 1.5 vs 21 ± 13.6 days, *P* = 0.01), DMV (1.9 ± 1.1 vs 13.3 ± 5.3 days *P* < 0.001), and chest tube duration (5.6 ± 1.2 vs 16.8 ± 9.8 *P* = 0.001) were all significantly lower in the operative group. The Constant–Murley score in the operative group was significantly higher (87.6 ± 5.4 vs 74.6 ± 9.8, *P* = 0.01). There were no complications regarding the rib fractures and only the two patients of whom the clavicle fracture was treated conservatively developed a non-union which required intervention. In the conservative group there were three patients who developed a pneumonia and two patients who had a bacteremia. The MINORS score of this comparative study was 18 (Online Appendix 2).

## Discussion

In this systematic review, an overview of all available literature on patients with concomitant clavicle and rib fractures was provided. Five studies on three different study populations showed that these two injuries were closely related in polytrauma patients [25–29]. In patients who suffered a blunt chest trauma, 18.6% had combined clavicle fractures and rib fractures.

Among polytrauma patients with clavicle fractures, there were approximately twice as much patients with rib fractures (56–60.6%) as compared to patients without clavicle fractures (29%). Vice versa in patients with multiple rib fractures or flail chests, clavicle fractures were present in 14–20.4%, which was approximately two to three times more often as compared to patients without rib fractures. Furthermore, clavicle fractures were seen more frequently in patients with rib fractures in the upper part of the thorax and the percentages of clavicle fractures increased with each additional fractured rib. Two studies reported on treatment of patients with clavicle fractures and rib fractures [30, 31]. One case series described 11 patients with clavicle fractures and flail chests with operative treatment for both injuries, who all had complete union of the fractures without complications [30]. One case series compared operative and conservative treatment in patients with clavicle and rib fractures [31]. Operative treatment of the injuries was found to significantly reduce ILOS, DMV, and chest tube duration. The Constant–Murley score was significantly better in patients who had operative treatment and no complications were reported after surgery.

Patients who sustain combined clavicle and rib fractures can be treated in four different ways; i.e., operative treatment of both injuries, operative treatment of the clavicle fracture only, operative treatment of the rib fractures only or conservative treatment of both injuries. Currently, there is no evidence on what treatment is most beneficial for patients with both injuries, while both isolated injuries and their treatment options have been thoroughly investigated in the past decade. A systematic review showed that in patients with flail chests, rib fixation led to shorter ILOS and DMV, lower pneumonia and mortality rates and less need for tracheostomy [18]. For patients with non-flail multiple rib fractures, similar significant outcomes of rib fixation were not yet reported. However, there is a trend towards operative treatment of patients with multiple displaced rib fractures as well, as an online survey showed that rib fixation was considered indicated for most patients with non-flail displaced rib fractures [32]. Also, a recent trial on patients with non-flail multiple rib fractures found that these patients could also benefit from rib fixation, as de numeric pain score after two weeks was shown to be significantly lower after rib fixation compared to after conservative treatment [16]. Furthermore, a good quality of life at least one year after surgery and adequate pulmonary function were seen after rib fixation, in both flail chest and non-flail multiple rib fracture patients [34, 35]. An extensive retrospective cohort study on the effect of rib fixation in patients with isolated thoracic injuries with rib fractures also showed that rib fixation was significantly associated with lower mortality rates, yet this association was not analyzed separately for patients with flail chests or non-flail rib fractures [36]. Several indications for rib fixation have been established, such as flail chests, reduction of pain and disability, chest wall deformity, respiratory failure, non-union, and open rib fractures [6, 32, 33]. Despite these indications, the exact group of patients who benefit most from rib fixation, while minimizing the risks of surgery, remains ambiguous. Isolated clavicle fractures can mainly be treated conservatively, although in some cases of severe displacement or comminution there is an indication for operative treatment as well [20, 21].

As the indications for operative treatment of the combined injury remain unknown, treatment varies between hospitals. Michelitsch et al. retrospectively analyzed patients who underwent rib fixation and reported that in cases of a concomitant ipsilateral clavicle fracture, this fracture was fixated first according to protocol [37]. Operative treatment of the rib fractures was still performed when patients could not be weaned from ventilation, or when there was a volume decrease or deformity of the thorax, and in cases of a significant flail chest. Langenbach et al. investigated the importance of a concomitant clavicle fracture in patients with rib fractures and reported that in patients with stable rib fractures combined with non-displaced clavicle fractures, both injuries were managed conservatively [12]. In cases of unstable but non-displaced rib fractures combined with a displaced clavicle fracture, the clavicle fracture was fixated and the ribs were additionally fixated only if there were relevant symptoms or constraints of the respiratory system. In patients with unstable displaced rib fractures and displaced clavicle fractures, both injuries were treated operatively.

There may be an indication for operative treatment of the clavicle fracture in patients with upper rib fractures if the clavicle could provide any stability to the upper chest wall. However, the role of the clavicle in supporting chest wall integrity has not yet been described in current literature. Previously, it has been described in what extent the clavicle obtains stability from the chest wall. There are two studies that found that rib fractures were associated with progressive displacement of a midshaft clavicle fracture, with an increasing risk of progressive displacement with each additional rib fracture [13, 14]. These results suggest that stability of the clavicle also in part depends on support of the chest wall. Taken these considerations into account, it could be reasoned that in cases of combined clavicle and rib fractures, at least one of those injuries, or perhaps both depending of the severity of the fractures, should be treated operatively. It could be argued that a concomitant clavicle fracture worsens pain induced breathing problems caused by rib fractures. Fixation of the relatively superficial clavicle might, therefore, be an easier intervention to restore stability or reduce pain as compared to rib fixation. Furthermore, fixation of a clavicle fracture enhances early mobilization which could lead to better outcomes. However, these speculations should be investigated in future studies. The main limitation of this study is the scarcity of studies reporting on patients with clavicle fractures and rib fractures. Second, the two studies on treatment described limited numbers of patients. The case series by Solberg et al. is the only study that compared patients with combined injuries who were treated operatively with patients who had conservative treatment and reported promising results in favor of operative treatment [31]. Nonetheless, no conclusions could be drawn on whether these improved outcomes where caused by fixation of the clavicle, or fixation of the ribs, or both. Third, it remains unknown whether this combined injury is also affected by a concomitant scapula fracture. Last, there could have been a publication bias.

Clavicle fractures and rib fractures are closely related in polytrauma patients and among patients who suffered a blunt chest trauma almost a fifth sustain both injuries. Based on the scarce literature, all recommendations on treatment remain speculative and definitive conclusions could not be drawn on treatment of patients with concomitant clavicle and rib fractures. Future research should further address the considerations that were discussed in this systematic review and investigate indications for and outcomes of operative treatment of patients with combined clavicle fractures and rib fractures. Also, biomechanical studies on this combined injury are needed to further understand the consequence of this injury on chest wall stability. Herewith, the role of the scapula should also be addressed.

## Supplementary Information

Below is the link to the electronic supplementary material.

Supplementary file1 (PDF 63 KB)

Supplementary file2 (XLSX 14 KB)

## Data Availability

Data were derived from published articles.
