# Supplementary material for: Epidemiology of combined clavicle and rib fractures: a systematic review
Source: Eur J Trauma Emerg Surg. 2021 Jun 1;48(5):3513–20. doi: 10.1007/s00068-021-01701-4 (PMC9532289; doi:10.1007/s00068-021-01701-4)
Supplement: Supplementary file 1 — Supplementary file1 (PDF 63 KB) [file 68_2021_1701_MOESM1_ESM.pdf]

Appendix 1. Search terms

MEDLINE:

(clavicle[MeSH] OR clavic* OR (collar AND bone))

AND

((“rib fractures”[MeSH] OR ((rib OR costal)) OR (((chest OR thoracic) AND wall)) AND (fractur* OR injur*)))

OR

(costoclavicular AND (fractur* OR injur*))

EMBASE:

(clavic*:ti,ab OR (collar:ti,ab AND bone:ti,ab))

AND

(rib:ti,ab OR costal:ti,ab OR ((chest:ti,ab OR thoracic:ti,ab) AND wall:ti,ab)) AND (fractur*:ti,ab OR injur*:ti,ab)

OR

(costoclavicular:ti,ab AND (fractur*:ti,ab OR injur*:ti,ab))

CENTRAL:

(clavic* OR (collar AND bone))

AND

((rib OR costal) OR ((chest OR thoracic) AND wall) AND (fractur* OR injur*))

OR

(costoclavicular AND (fractur* OR injur*))
